# Deep Learning Scene Recognition Method Based on Localization Enhancement

**DOI:** 10.3390/s18103376

**Published:** 2018-10-10

**Authors:** Wei Guo, Ran Wu, Yanhua Chen, Xinyan Zhu

**Affiliations:** State Key Laboratory of Information Engineering in Surveying, Mapping and Remote Sensing, Wuhan University, Wuhan 430079, China; guowei-lmars@whu.edu.cn (W.G.); wuran1994@whu.edu.cn (R.W.); xinyanzhu@whu.edu.cn (X.Z.)

**Keywords:** scene recognition, deep learning, indoor positioning, signals of opportunity

## Abstract

With the rapid development of indoor localization in recent years; signals of opportunity have become a reliable and convenient source for indoor localization. The mobile device cannot only capture images of the indoor environment in real-time, but can also obtain one or more different types of signals of opportunity as well. Based on this, we design a convolutional neural network (CNN) model that concatenates features of image data and signals of opportunity for localization by using indoor scene datasets and simulating the situation of indoor location probability. Using the method of transfer learning on the Inception V3 network model feature information is added to assist in scene recognition. The experimental result shows that, for two different experiment sceneries, the accuracies of the prediction results are 97.0% and 96.6% using the proposed model, compared to 69.0% and 81.2% by the method of overlapping positioning information and the base map, and compared to 73.3% and 77.7% by using the fine-tuned Inception V3 model. The accuracy of indoor scene recognition is improved; in particular, the error rate at the spatial connection of different scenes is decreased, and the recognition rate of similar scenes is increased.

## 1. Introduction

Innovative positioning technologies and wireless networks promote the development of the indoor positioning system [1,2]. At the same time, with the improvement of hardware and developments in behavior calculation, more and more intelligent devices are being used in scientific research and real-world scenery [1]. The acquisition of real-time location information on mobile devices has become an indispensable element of intelligent devices [3]. Since the mobile terminal contains built-in physical devices, such as vision and multi-sensors, it has become a new type of scene perception [4] and communication [5] platform for image acquisition and decision-making. Mobile terminals can provide basic communication information and perception for positioning and scene recognition technology [6]. In indoor positioning, which uses the mobile terminal as the development platform, Signals of Opportunity (SoOP) [7], such as Wi-Fi or Bluetooth signals, are not specifically built to be positioning signals [8], but these signals have become a widely-researched topic of indoor positioning, because they are easy to build up and are cost-effective. Chen et al. [3] achieved positioning accuracy of up to 2–5 m by using the fusion signal of the built-in sensor, Wi-Fi, and magnetic field. This indicates that SoOP can work in combination with other positioning sources to provide accurate, reliable, and real-time positioning.

However, current research is mainly focused on the aspects of implementation or auxiliary positioning [9]. There is insufficient usage of the relationship between positioning information and the scenery semantic, for example, when users are in an indoor environment holding their mobile devices, through the positioning signals, we can obtain the positioning information that relates to the scenery’s geometry structure; then, we can obtain the semantic information of the current position by combining the positioning information with the elements that describe the scene (such as the indoor map). Therefore, in this paper, we will discuss a method to mine the positioning information of SoOP, and use that information in scene recognition.

The traditional method [10] of scene recognition mainly focuses on the description of image features. Differing from outdoor scenery, indoor scenery is relatively complex with various layouts and decorations [11]; therefore, indoor scene recognition should take both the local features and the global features of the image into consideration. In 2009, Quattoni et al. [12] designed a scene recognition model called the Spatial Envelop Model, which combines local and global descriptors of the image. In other studies, Swadzba et al. used 3D geometric models [13] and 2D global GIST features to recognize indoor scenes, and Lanzebnik et al. designed a new type of visual descriptor for image features [14]. Since these traditional methods cannot achieve high classification accuracy, they cannot satisfy the processing of large-scale data.

With the growth in the quantities of image data and categories to be classified, a method based on deep learning is introduced into the scene recognition task, such as Place-CNN, which is trained by the large-scale dataset Places2 [15] to focus on scenery issues. After AlexNet [16] won the LSVRC (large-scale visual recognition challenge), deep learning has been more and more frequently applied to scene recognition tasks and daily life, such as project tensorflow-for-poets: Inception-V3 [17], which has realized a relatively high accuracy on real-time scene recognition. The feature extracted by the Inception-V3 neural network is more discriminative and accurate than those for manual fabrication. There are also ways to deal with recognition tasks with several different forms of information, like Nguyen et al. [18] did, where they concatenated the underlying features of three deep convolutional neural networks to come up with the final decision. Tang et al. developed a CNN model, which learns the features in the convolutional neural network in multi-stage [19].

However, the existing methods are still unable to solve the task of scene recognition in indoor environments. The indoor scenery has rich and disordered indoor environment features, providing a deep learning method with a considerable source of training image data. Additionally, this also causes problems in image processing. The image from the mobile device inside the indoor environment has a similarity between categories (such as office and machine room), and differences within the same category (such as administrative office and teaching office), and the label classification contains strong subjectivity. The aforementioned points make the accuracy of indoor scene recognition lower than that of outdoor scene recognition. Further, all the methods above rely on the image feature information unilaterally, which requires massive data to guarantee the quality of model training; furthermore, this may limit the generalization ability of the model. Considering that indoor scenes have complex spatial geometry relationships, the feature information of indoor scenery is difficult to extract from the image.

According to the problem mentioned above, to build up the relationship between the positioning information and the scenery, and get rid of the limitation where deep learning-based scene recognition only relies on image information for learning and classification, in this paper, a neural network is designed to combine the positioning and image features, and improve the accuracy of indoor scene recognition by taking advantage of real-time images and positioning information. In this paper, we use the fine-tuned Inception-V3 model to extract image features from the scenery image, and we describe the non-image features using the probabilistic features of scene recognition within the range of positioning error. By connecting the image and non-image features, we built a scene recognition network based on indoor SoOP enhancement. Compared to the scene recognition method based solely on image or positioning features, the proposed model has a significant improvement in recognition accuracy, and obtains a high recognition accuracy and good generalization ability when trained with a small amount of training data.

## 2. Related Work

There is a large number of studies related to scene recognition and to deal with this problem, large datasets have emerged. In order to understand the scene-centered way of human recognition, reference applied the gist of a scene for improving scene image recognition by the mean of global image features [20]. A popular way of bridging the semantic gap in scene recognition is semantic modeling, in [21], a manifold regularized deep architecture is designed to get good performance. In fact, data is also an important direction in scene recognition tasks. In the case of sufficient data, even the simplest model algorithm can be used and get a pretty good classification result. There is a diverse set of 67 indoor scenes for indoor scene classification, which contains 15,620 images [12]. Based on deep learning, the places [22] provide tremendous indoor and outdoor scene images and the reference trained models for learning deep features of scene-centered images. The Places dataset contains seven million tagged images, a total of 476 categories, are the largest image dataset including number of scenes and locations. Additionally, the most famous database named ImageNet [23], hierarchical and diversified, is widely used, which can be applied to object recognition, image classification, and automatic target clustering, etc. Due to these large databases, ImageNet-trained deep features can actually have success in different types of work [24]. Likewise, our work is to transfer learning the well pre-trained model based on large Imagenet datasets and then feed the model with our own dataset for the purpose of tackling the problems of our work.

Currently, some work of computer vision has realized the importance of utilizing geographic information. One focus is on the research of visual location. In [25], the image geolocalization can be greatly extended by the proposed cross-view feature translation method, which means to enrich attributes of geo-tagged imagery. In addition to the image itself, the methods [26] consider the digital elevation models with target mountain terrain to exploit the visual location. However, the other focus, putting geographic information into visual tasks, like image classification, is more similar to our study. In [27], to estimate priors of spatio-temporal classes at irregular and biased locations significantly improves performance. In [28], the encoding of GPS coordinates are extracted and merged into a convolutional neural network for image classification and it achieves improvement in the mean average precision. In [29], a geographical information system (GIS) assists in object detection, which computes GIS priors of the visible objects in the image to detect the final bounding boxes. In [30], the problem of object recognition is tracked with image appearance and geo-services positioning information on mobile devices. In [31], through picture metadata, the method uses the nonvisual context information (season and location) to achieve scene understanding in customer photos. In [32], they deeply study three spatial contexts to improve image classification.

Compared with previous approaches, this paper explicitly exploits the combination of positioning information and image information to improve scene recognition. We are interested in the performance of two types’ combination. By transfer learning, we learn and extract image features; by simulative positioning error, we design two positioning features representing the scene attribute. We assess two scene places’ recognition accuracy in different categories under four schemes. This can provide different situations which improve the scene recognition.

## 3. Feature Fusion Algorithm

**Figure 1:**
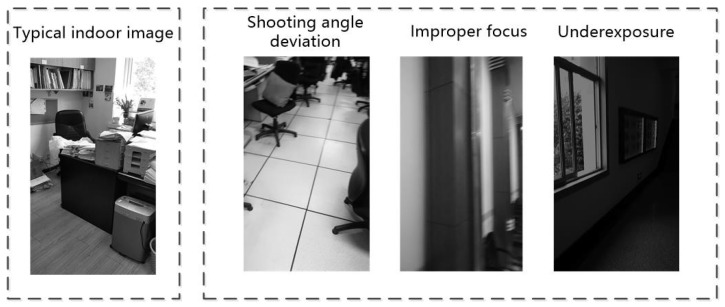
Comparison between normal indoor image and real-time image from a mobile device.

Indoor scenes are different from outdoor scenes, which have a wide range of background elements with relatively stable features. Based on the convolutional neural network [16], to obtain more accurate scene prediction results, we only need to extract the most prominent and typical background information from the outdoor scenery. The indoor scene has a large number of foreground objects, and even for the same kind of scenery, due to the differences in interior decoration, style, and other factors, the features are very diverse. Therefore, it is difficult to obtain high recognition accuracy by using the convolutional neural network with limited typical indoor scenery image for training. When considering real-time images captured by mobile devices, due to the high randomness and disturbance in the shooting angle, focus, and exposure (as Figure 1 shows), the captured image will have a large amount of random noise, and its features are much weaker than those of a typical indoor image, which makes it difficult for convolutional neural networks trained with typical indoor scene images to extract prominent scenery features from this type of image.

Compared with image information, positioning information in the indoor scene cannot directly offer scene features, but the spatial relationship with each scene can be reflected through certain calculations, along with the indoor map. In this way, we can also judge the category of the scene at the current location. However, as indoor positioning accuracy is difficult to guarantee in situations of weak positioning signals or few signal sources, there will be a high risk of inaccuracy in the scenery prediction by using this information directly, especially in the area of multi-scene junctions and scene boundary areas. Thus, it is difficult to guarantee a high accuracy of indoor scene recognition when indoor image information or positioning information is provided alone.

**Figure 2:**
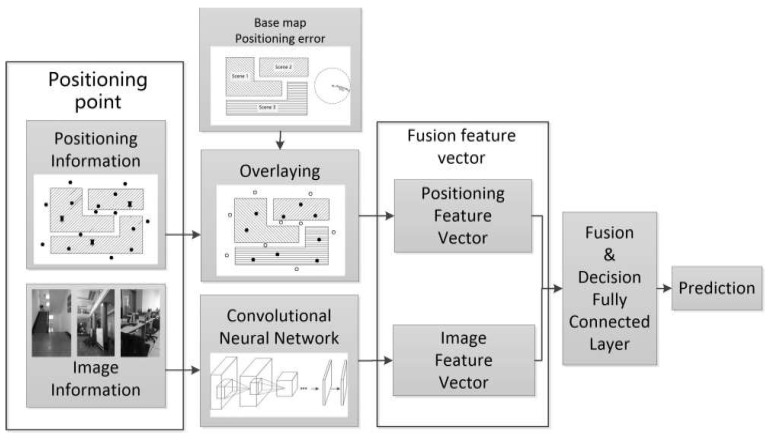
The algorithm flow chart for deep learning scene recognition method based on localization enhancement.

Based on the above analysis, this paper puts forward a deep learning scene recognition method based on localization enhancement. Here, we extract image and positioning features from the scenery image and positioning data from positioning points, then use the method of deep learning to train the optimal feature fusion strategy for the fusion operation of these two features; finally, the fusion feature is used to obtain the scenery prediction results. The algorithm is shown in Figure 2. We extract the scene level feature vector of the scene image (labeled as $V_{image}$) by using a convolutional neural network that is fine-tuned to the scene studied, and extract the positioning feature vector by overlaying the operation using the positioning information of the location site with the estimated error and the indoor scene base map. Further, the image feature vector and the positioning feature vector are combined into one feature, denoted as follows:

(1) $$V_{fuse}=\left\{V_{image}\vdots V_{positioning}\right\}$$

The combined feature vector $V_{fuse}$ is transformed by the feature fusion fully connected layer, then the output, called the fusion vector, is processed by the final decision fully connected layer and the output is the final prediction result.

### 3.1. Image Feature Vector

**Figure 3:**
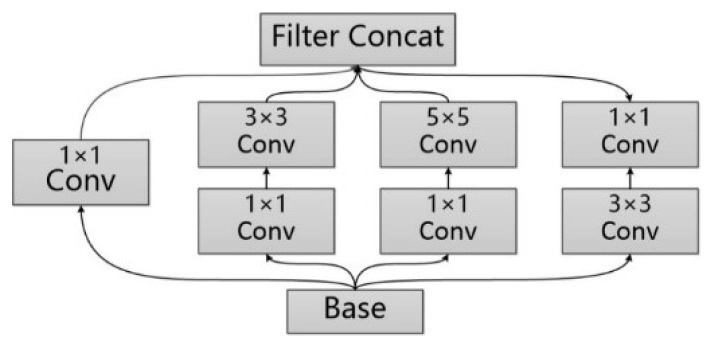
Module of Inception.

GoogleNet [33] improves the prediction result of the neural network by increasing the depth and width of the network. The depth of the network represents the number of layers the network has, and the width of the network represents the number of neurons in every layer. In order to solve the problem of overfitting of the network and the cost surging of model training, GoogleNet is added with the Inception structure (as shown in Figure 3). In the Inception V1 [33] version, a 1 × 1 convolution kernel is introduced into the structure to make the feature map thinner; for Inception V2 [34], on the one hand, the BN [35] layer is introduced to make the output parameters of every layer normalized into the Gaussian distribution of N(0,1), in order to lower the risk of overfitting, and on the other hand, the method of replacing the 5 × 5 convolution kernel with two 3 × 3 convolution kernels, which is used in VGG [36] net, is introduced into the Inception structure for further reduction of the number of parameters; the Inception V3 [17] version introduced the concept of convolution decomposition, that is, decomposing the n × n convolution kernel into two one-dimensional 1 × n and n × 1 kernels, to speed up computing of the network and increase the depth of the network.

There are foreground objects of different scale, size, and scope in an indoor scene image, making it difficult for a single type of convolution kernel to fully perceive these different scene features. However, the model construction method of the Inception module with different size of the paralleled convolution cores is suitable for solving the feature extraction task of such complex scene images. Therefore, we select the Googlenet model with Inception V3 structure as the image feature vector extraction module. By inputting the scene image obtained by the mobile device into the module, we can extract the scene level image feature vector, which is used to reflect the image feature of the current scene of the mobile device. The image feature is involved in the subsequent feature fusion layer, and conducts the fusion operation in combination with the positioning feature vector.

In order to avoid overfitting due to the module learning to the random noise from mobile device images, we will use a small number of typical scene images in the fine-tuning [37] process of the Inception V3 module. The typical scene images should have prominent scene features, significant difference between categories, rich features in every category, and a nearly even quantity of images in every category. After several layers of convolution, pooling and other operations on the image data, the Inception V3 module will output a bottleneck value tensor of [1, 2048] size, which reflects the underlying features in the output of the convolution operation on the image. The process of fine-tuning can be seen as the process of transforming the bottleneck value feature into the scene level image feature, and its essence is to adjust the shape and parameter of the fully connected layer behind the convolution operation, so as to make the final output results of the model adapt to the problem studied.

According to $N_{category}$, which represents the number of scene categories of the studied area in this paper, all parameters connected to the activation of the last convolution layer of Inception V3 are replaced with the fully connected layer with $\left(2048\times N_{category}\right)+N_{category}$ parameters, and the new layer outputs the final result through Softmax operation. The parameters of the fully connected layer are trained by using the typical image dataset collected from the studied scene in this paper, while the original parameters of all convolution layers are maintained. After the training is completed, the parameters of the fully connected layer are fixed and saved with the original convolution layer parameters as a new Inception network model.

### 3.2. Positioning Feature Vector

As the positioning points in the scene are affected by the signal intensity and number of signal sources, the positioning result often has some uncertainty, which is manifested as the random distribution of the positioning points within a certain error range. Therefore, the scene prediction result obtained by the positioning information is only a probability value instead of a certain prediction result. To conduct the prediction by making full use of the geometric location information of the positioning points in the scene and the feature information of the relationship with the surrounding scene, in this paper, we will use the location features and the features of the relationship with all surrounding scenes to describe the positioning feature of the positioning points.

The location scene feature mainly represents the semantic representation of the plane coordinate (x,y) of points in the scene, that is, the scene category where the positioning point is located. Considering that the positioning point may be located outside the scene boundary due to an error, it is necessary to see this situation as a specific scene category. This feature can be expressed in the form of a one-hot encoding in {0,1} distribution, with the shape of $\left[1,N_{category}+1\right]$, where $N_{category}$ represents the number of scene categories studied. By overlaying the positioning points with the scene base map, we assigned 1 to the element whose index corresponds to the category index number of the scene that the point falls into, and set the remaining elements in the one-hot vector to 0. If the positioning point is outside the scene, the first element is set to 1 and the rest to 0, as follows:

(2) $$V_{location}=\{\begin{array}{c} \left\{1\vdots \left\{V_{1}\;\cdots V_{N_{category}}\right\}\right\},\forall V_{i}=0\;\left(a\right)\\ \left\{0\vdots \left\{V_{1}\;\cdots V_{N_{category}}\right\}\right\},V_{k}=1\;\left(b\right) \end{array}$$

where *i* and *k* represent the scene category index, and Equation 2a represents the situation where the point belongs to no category, and Equation 2b represents the situation when the point is located in the *k*th type of scene category.

**Figure 4:**
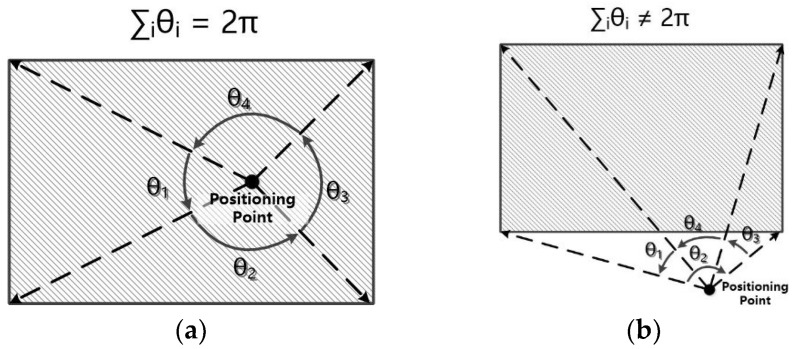
Positioning conditions of location point. (**a**) Inside the scene; (**b**) outside the scene.

The algorithm of overlaying the positioning points and scene base map can be equivalent to the method of judging whether the point is inside a polygon. There are several popular algorithms to solve this, including discriminating by the sum of area, discriminating by sum of angle, and the leading ray method. In this paper, we use the sum of area for discrimination. By saving the scene base map in the format of GeoJSON, we can use the type of polygon to save the boundary of scenes, and the points encoded around the outer boundary of the scene are anti-clockwise. A positioning point $P_{o}$ can form vector $\vec{P_{o}P_{i}}$ and $\vec{P_{o}P_{i+1}}$ along with any two sequential points $P_{i}$ and $P_{i+1}$, and we can obtain the angle between these two vectors using the formula $\theta =\cos^{-1}\frac{\vec{P_{o}P_{i}}\cdot \vec{P_{o}P_{i+1}}}{\left\|\vec{P_{o}P_{i}}\right\|\left\|\vec{P_{o}P_{i+1}}\right\|}$. Sum up all the angles formed by the positioning point and all the possible combinations of sequential points; the point is located inside the scene if the sum equals 2π, otherwise the point is located outside the scene. In addition, we need to consider the situation where some scenes have inner boundaries, and in this situation if the sum of angles equals 2π, the point is located outside the boundary, as shown in Figure 4. The algorithm’s flow chart is shown in Algorithm 1.

**Algorithm 1** Feature vector V_location**Input:** positioning point $P_{o}\left(x_{o},y_{o}\right)$; collection of all scene boundaries $D=\left\{D_{1},\;D_{2},\;\cdots ,\;D_{n}\right\}$.**Procedure:** function $\mathrm{GetSceneCategory}\left(P_{o},\;D\right)$**1:** feature vector $V_{\mathrm{location}}\leftarrow {\left\{0\right\}}^{N_{\mathrm{category}}+1}$**2:** For each scene $D_{i}=\left\{T_{1},\;T_{2},\;\cdots \right\}$ in collection D**3:**  For each polygon $T_{i}=\left[\left(x_{1},y_{1}\right),\left(x_{2},y_{2}\right),\cdots ,\left(x_{n},y_{n}\right),\left(x_{1},y_{1}\right)\right]$ in $D_{i}$**4:**   For each pair of sequential points $P_{i},P_{i+1}$ in $T_{i}$**5:**    calculate the angle $\theta_{i}$ between $\vec{P_{o}P_{i}}$ and $\vec{P_{o}P_{i+1}}$**6:**    sum up angles $\theta_{\mathrm{Sum}}\leftarrow \theta_{\mathrm{Sum}}+\theta_{i}$**7:**   End For**8:**   If $T_{i}$ is the outer boundary and $\theta_{\mathrm{Sum}}$ equals 2π**9:**    element $V_{k}\leftarrow 1$ in feature vector $V_{\mathrm{location}}$, where k represents the category of scene $D_{i}$**10:**   Else If $T_{i}$ is the inner boundary and $\theta_{\mathrm{Sum}}$ equals 2π**11:**    element $V_{k}\leftarrow 0$ in feature vector $V_{\mathrm{location}}$, where k is the category of scene $D_{i}$**12:**   End If**13:**   End For**14:** End For**15:** If elements of feature vector $V_{\mathrm{location}}$ all equal to 0**16:**  element $V_{0}\leftarrow 0$ in feature vector $V_{\mathrm{location}}$, means the point locates outside all the scenes**17:** End If**Output:** feature vector $V_{\mathrm{location}}$

The feature of the relationship between the positioning point and the surrounding scenes is related to the current positioning error. The larger the error is, the larger the region of scenes related to the positioning point, and the more complicated the scene category involved. In particular, when the point is near the junction of multiple scenes, the uncertainty of scene prediction is greater, so the relationship between the positioning with error and surrounding scenes needs to be expressed in some way. The positioning error can be estimated by using parameters such as the intensity of the positioning signal and the number of available signals while, in the experiment in this paper, the positioning error in the indoor scene is simulated by using the preset parameters. By overlaying various scene boundaries with the error circle whose center is the positioning point and radius equals the positioning error, we can measure the area of intersection between scenes and the error circle. The value of the area can reflect how significant the relationship between the current positioning point and the scene is. The more significant the relationship is, the more likely this positioning point is located inside this scene. By organizing the value of each area into a $\left[1,N_{category}\right]$ sized tensor and normalizing the element values to the range of [0, 1], the significance of the relationship with the scene is expressed by the size of the probability values, such as:

(3) $$V_{relation}=\left\{S_{i}/\sum_{1}^{N_{category}}S_{i}\right\}$$

where $S_{i}$ represents the area of intersection of the *i*th scene category and the error circle.

**Figure 5:**
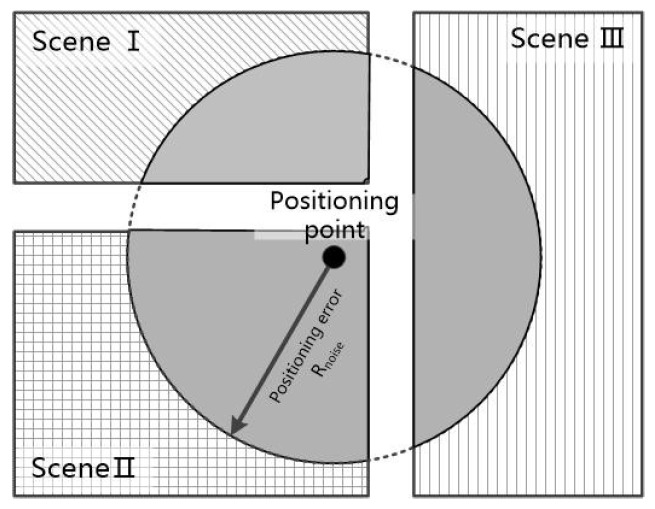
The overlap of scene and error circle.

The calculation of the intersection area of the multi-boundary scene and the error circle can be divided into the calculation of the intersection area of the triangle and circle. As shown in Figure 5, every positioning point, say $P_{o}$, can form a triangle $\Delta P_{o}P_{i}P_{i+1}$ with any two of the sequential points in the boundary, such as $P_{i}$ and $P_{i+1}$. In this way, we can calculate the intersection areas of the error circle with each triangle corresponding to each border line; finally, the intersection area of the error circle and the scene boundary is the sum of the areas above. It should be noticed that, according to the encoding rules of GeoJSON, the outer boundary of polygons is encoded counterclockwise, while the inner boundary is encoded clockwise, so the value of area calculated by the outer boundary is positive in the method of the vector’s cross product, and by the inner boundary, the result is negative. The detailed algorithm flow chart is shown in Algorithm 2.

**Algorithm 2** Feature vector V_relation**Input:** positioning point $P_{o}\left(x_{o},y_{o}\right)$; pre-set error R; collection of all scene boundaries $D=\left\{D_{1},\;D_{2},\;\cdots ,\;D_{n}\right\}$.**Procedure:** function $\mathrm{GetRelation}\left(P_{o},\;D\right)$**1:** feature vector $V_{\mathrm{relation}}\leftarrow {\left\{0\right\}}^{N_{\mathrm{category}}}$**2:** For each scene $D_{i}=\left\{T_{1},\;T_{2},\;\cdots \right\}$ in collection D**3:**  For each polygon $T_{i}=\left[\left(x_{1},y_{1}\right),\left(x_{2},y_{2}\right),\cdots ,\left(x_{n},y_{n}\right),\left(x_{1},y_{1}\right)\right]$ in scene $D_{i}$**4:**   For each pair of sequential points $P_{i}$,$P_{i+1}$ in $T_{i}$**5:**    If segment $P_{i}P_{i+1}$ is inside the error circle**6:**     sum up the area $S_{\mathrm{Sum}}\leftarrow S_{\mathrm{Sum}}+S_{\Delta P_{o}P_{i}P_{i+1}}$**7:**    Else If segment $P_{i}P_{i+1}$ is outside the error circle**8:**     respectively calculate the intersection $P_{i}^{\prime }$ and $P_{i+1}^{\prime }$ of $P_{o}P_{i}$, $P_{o}P_{i+1}$ and the error circle**9:**     sum up the area $S_{\mathrm{Sum}}\leftarrow S_{\mathrm{Sum}}+S_{{\mathrm{sector}\;P}_{i}^{\prime }P_{o}P_{i+1}^{\prime }}$**10:**    Else**11:**     calculate the intersection $P_{c}$ of $P_{i}P_{i+1}$ and the error circle**12:**     calculate the intersection $P_{i}^{\prime }$ (or $P_{i+1}^{\prime }$) of $P_{o}P_{i}$ (or $P_{o}P_{i+1}$) and the error circle**13:**     sum up the area $S_{\mathrm{Sum}}\leftarrow S_{\mathrm{Sum}}+S_{\Delta P_{o}P_{c}P_{i+1}^{\prime }}+S_{{\mathrm{segment}\;P}_{c}P_{o}P_{i}^{\prime }}$**14:**    End If**15:**   End For**16:**   element $S_{i}\leftarrow S_{i}+S_{\mathrm{Sum}}$ in feature vector $V_{\mathrm{relation}}$, where k is the category of scene $D_{i}$**17:**  End For**18:** End For**19:** normalize elements in feature vector $V_{\mathrm{relation}}$**Output:** feature vector $V_{\mathrm{relation}}$

The location scene feature vector and the relation vector between the positioning point and the surrounding scene are merged to form the positioning feature vector, sized as $\left[1,2*N_{category}+1\right]$, as follows:

(4) $$V_{positioning}=\left\{V_{location}\vdots V_{relation}\right\}$$

### 3.3. Neural Network Model Design

**Figure 6:**
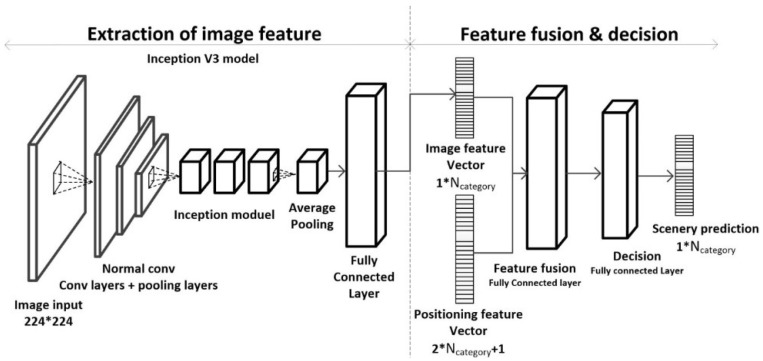
Model chart for the feature fusion model.

The feature fusion model designed in this paper is mainly composed of the Inception V3 convolution processing image feature extraction module, and the feature fusion and decision module (as shown in Figure 6). The Inception V3 convolution based image feature extraction module is obtained through fine-tuning with typical scene images, as described in Section 3.1. The feature fusion and decision module is composed of a feature fusion fully connected layer and final prediction fully connected layer, and the parameters are obtained through the simulative localization and image training set.

The neural network requests two types of input data, one is the image data of the corresponding positioning point and the other is the positioning feature vector $V_{positioning}$, which is obtained through the localization data process in Section 3.2. The image will be processed into a one-dimension image feature vector sized as $\left[1,N_{category}\right]$ by the Inception V3 module’s convolution process, and the image feature vector will be combined with the positioning feature vector $V_{positioning}$ as a one-dimension vector $V_{fuse}$, sized as $\left[1,3*N_{category}+1\right]$, which will be the input in the latter process module. Through training a fully connected layer with ${\left(3\times N_{category}+1\right)}^{2}+3\times N_{category}+1$ parameters using the ReLU function as the activation function, we can turn the feature vector combined with the image feature vector and the positioning feature vector into the fused vector of image information and positioning information. Finally, we train a fully connected layer with $\left(3\times N_{category}+1\right)\times N_{category}+N_{category}$ parameters using a Softmax operation to output the final scene prediction result.

### 3.4. Model Training

The training process of the model mainly focuses on two major modules, namely, the image feature extraction module and the feature fusion and decision module. The processes are as follows:

- Fine-tune the Inception V3 model: We used the typical scene image set to fine-tune the pre-trained Inception V3 model and replaced the fully connected layer behind the previous convolution layer with a new fully connected layer whose input tensor has the dimensions [1, 2048] and output tensor with $\left[1,\;N_{category}\right]$ dimensions. The original parameters of the convolution layers are retained and the parameters of the new fully connected layer are updated via training.
- Training the feature fusion and decision layers: Using the processed positioning feature vector and the image feature vector from the fine-tuned Inception V3 module as the input data for the fully connected feature fusion layer, the output tensor propagates forward to the decision fully connected layer. Together with the ground-truth data, we can calculate the loss of training by the prediction result from the fully connected decision layer, and we use the training loss to update the parameters of the previous two fully connected layers.

In this paper, we built the feature fusion model base on the TensorFlow [38] framework, which is an open source artificial intelligence learning system developed by Google and is widely used in machine learning. The following hardware was used for training: CUP: 2.50 GHz Intel i5-7300HQ and GPU: GTX 1050 4 GB. The main parameters set in the training process are listed in Table 1:

## 4. Design of the Model Validation Experiment

### 4.1. Datasets

#### 4.1.1. Model Training and Validation Set

In this study, we prepared different training data sets for fine-tuning the Inception V3 model and training the feature fusion and decision layers. We divide the datasets into train dataset and evaluation dataset (85%, 15% respectively). For fine-tuning the Inception V3 model, we collected five classes in the 2nd floor of the laboratory, and each class has more than 600 images; eight classes in the railway station, and each class has more than 800 images, except the indoor channel with 600 images. For our proposed model, we collected 3000 images of the same classes in the 2nd floor of laboratory and 3500 images of the same classes in the railway station, according to the number of simulative positioning points.

We adopted a typical indoor image set for fine-tuning Inception V3. The dataset must have prominent scene features, significant difference between categories, rich features in every category, and the number of images in every category must be approximately equal. The dataset is used to train the Inception V3 model to function as the image feature extraction module. The aforementioned features of the dataset can ensure that the fine-tuned model is be sensitive to the important scenic features of the image captured using a mobile device and reduce the effect of noise in the image.

We used simulative positioning and the image dataset to train the feature fusion and decision layers. The dataset consists of two parts, the simulated point data and the corresponding image data. Together, they can simulate a given state of a mobile device in an indoor environment. The simulated point dataset is generated by adding random noise (in this experiment, the noise range is 5 m) to the appropriate positioning points that are chosen along the main roads of the scene. Each simulated point corresponds to the scene image that was collected from the exact point in the scene that it belongs to. The image must have strong randomness, which simulates the situation when the mobile device moves indoors. This randomness can be implemented in different ways, such as shooting angle, focus, and exposure.

**Figure 7:**
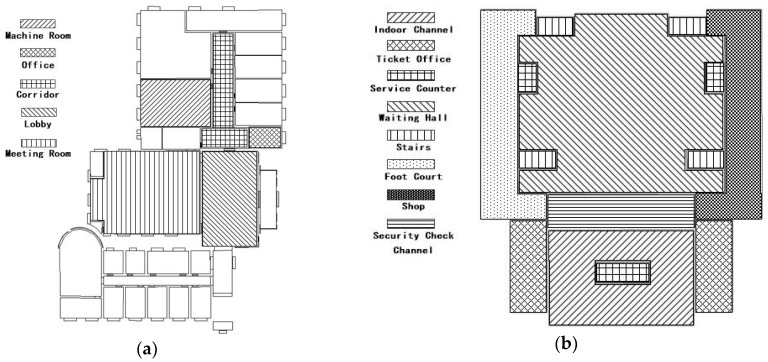
Experimental scene’s distribution map of model training and validation dataset. (**a**) 2nd floor of laboratory; (**b**) railway station.

In this paper, we chose the following two kinds of typical indoor scenes as the target for experiment: The second floor of the laboratory and the railway station. The laboratory scenery is classified into five categories, which includes a machine room, office, corridor, lobby, meeting room. The railway station scenery is classified into eight scene categories, namely, indoor channel, ticket office, service counter, waiting hall, stairs, foot court, shop, and security check channel. The scene distribution is shown in Figure 7.

#### 4.1.2. Validation Set for Generalization Ability

**Figure 8:**
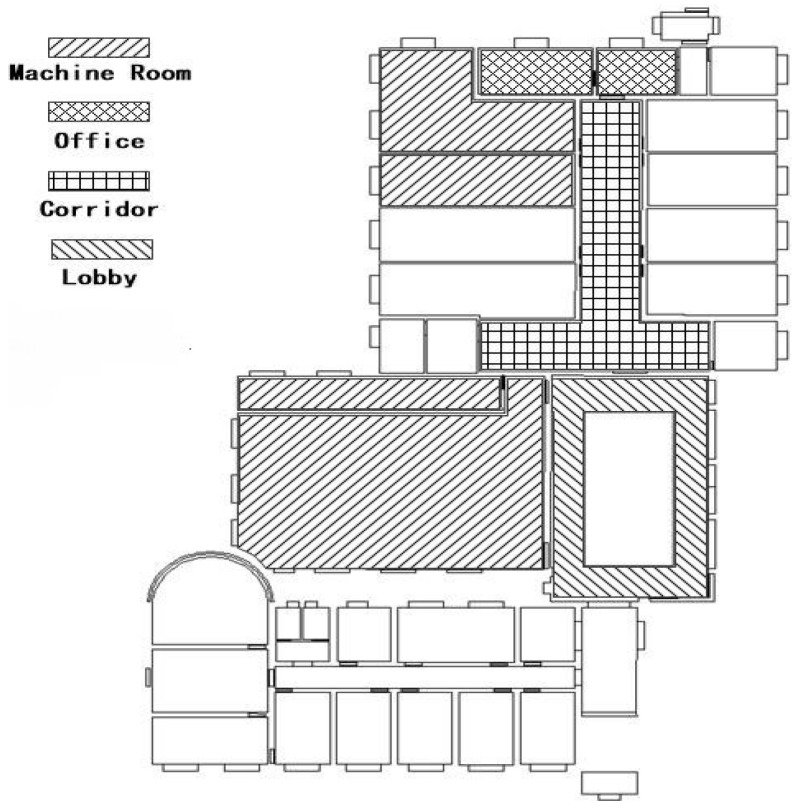
Distribution of the scene’s map for the generalization validation dataset.

In order to compare the generalization ability of the fine-tuned Inception V3 model and the feature fusion model, we selected the third floor of the laboratory with a category of scenery category similar to that of the second floor as the dataset for validation of the generalization. We prepared the simulative positioning and image set with the same range of noise as the experiment on the second floor, and we collected images from the third floor scenery including the machine room, office, corridor, and lobby. The distribution of scenery is shown in Figure 8.

### 4.2. Experimental Design

To compare the performance of the feature fusion model in this paper and the traditional convolution neural network in the indoor scene identification task, in the experiment was conducted to obtain the following four levels of comparison as shown in Table 2: Plan 1, in which the dataset of the second floor of the laboratory was used to fine-tune the Inception V3 model and to train the model designed in this paper. A subset of the data was used as a test set to test the identification performance of the two models. Plan 2 is similar to plan 1. We tested the identification performance of models with the same method in the railway station scenery. Plan 3, to determine the performance of this model with positioning points at different positioning accuracies, we constructed different positioning datasets with different positioning errors in the second floor of the laboratory scene while retaining the image information. We used the datasets of different positioning accuracy to test the performance of the models. In plan 4, to compare the generalization ability of the Inception V3 and the model in this paper, we selected the third floor of the laboratory as the experimental site. The third floor has scenery similar to that of the second floor. We then constructed the test set based on the procedure of construction for a simulative localization and image set and we tested the model trained based on the dataset of the second floor.

## 5. Experiment

### 5.1. Comparison Experiments on Prediction Performance

#### 5.1.1. The Result of Laboratory Scene

**Figure 9:**
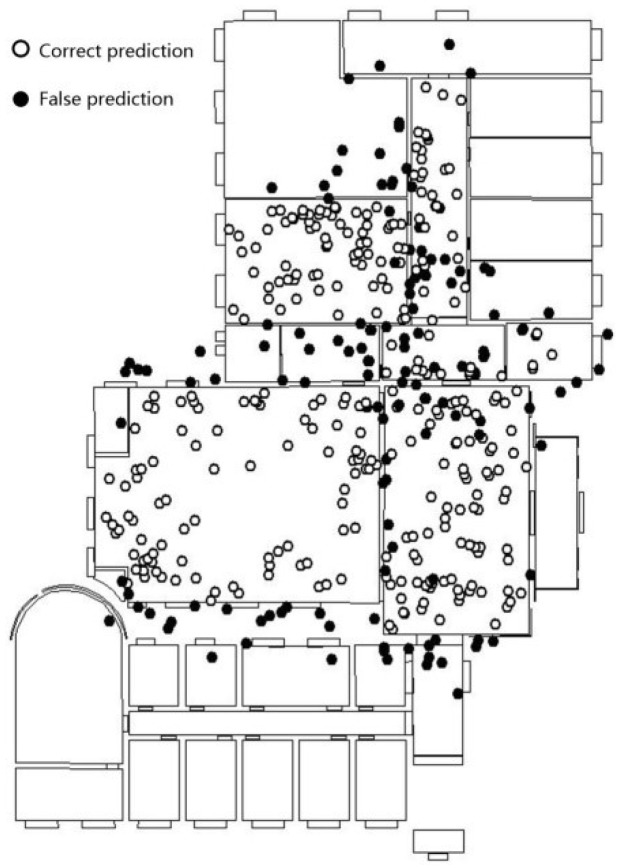
Overlapping result of location points in the 2nd floor of the laboratory.

For the laboratory scene, the fine-tuning procedure on the Inception V3 model achieves convergence after 2000 iterations, and the training of feature fusion and decision module achieves convergence after 10,000 iterations. The test set is 15% of the simulative localization and image data. Figure 9 shows the result of the overlapping of the positioning point of the test sample set and scene boundary (black spots are prediction error points). The percentage of the scene in the positioning point that is consistent with the real value is 69.0%. The error points are mainly concentrated near the scene boundary, especially at the intersection of the machine room, corridor, and lobby.

**Figure 10:**
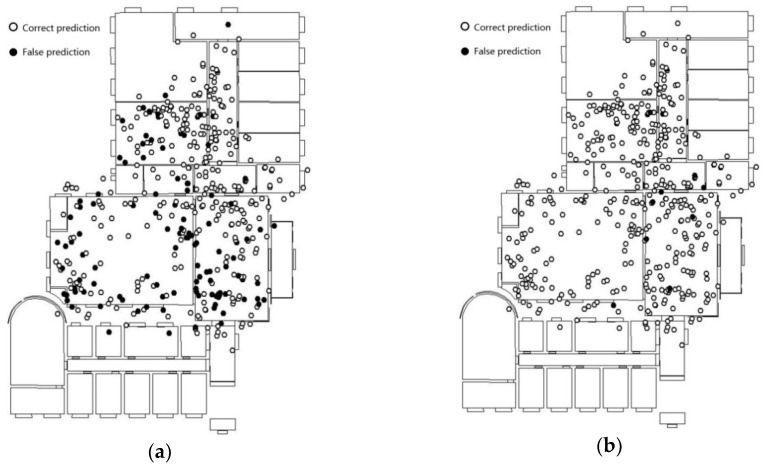
The prediction result in the 2nd floor of laboratory scenario. (**a**) Inception V3; (**b**) the feature fusion model.

The fine-tuned Inception V3 model is fed on the image dataset corresponding to the positioning points. The prediction results are as shown in Figure 10a. The prediction results of the feature fusion model are shown in Figure 10b.

The prediction accuracy of the fine-tuned Inception V3 model is 73.3%. The distribution of error points vary between scenes. For example, in the lobby scenes, the error points are mainly concentrated in the lower part of the scene map. In addition, Inception V3 yields good recognition results in scenes with relatively simple and singular layouts (such as the corridor). Since there are fewer foreground objects and a higher similarity in terms of background features, the image obtained by the mobile device is subjected to lesser disturbance in terms of shooting angle, focus, and exposure. Additionally, even the noise is relatively weak.

The accuracy obtained for the second floor of the laboratory using the feature fusion model is 97.0%. The error points near the boundary of the scene have generally been corrected compared with results of overlapping way as Figure 9. In particular, the prediction results for locations beyond the scene showed the most significant improvement. In contrast to the fine-tuned Inception V3 model, the feature fusion model that combines positioning and image features, is less-dependent on image features and extends the Eigenspace of positioning points. When the image quality is poor, results cannot always accurately reflect the scene feature; however, accurate prediction results can still be yielded. As a result, there is no significant difference in the distribution of error points in terms of the scene category.

**Figure 11:**
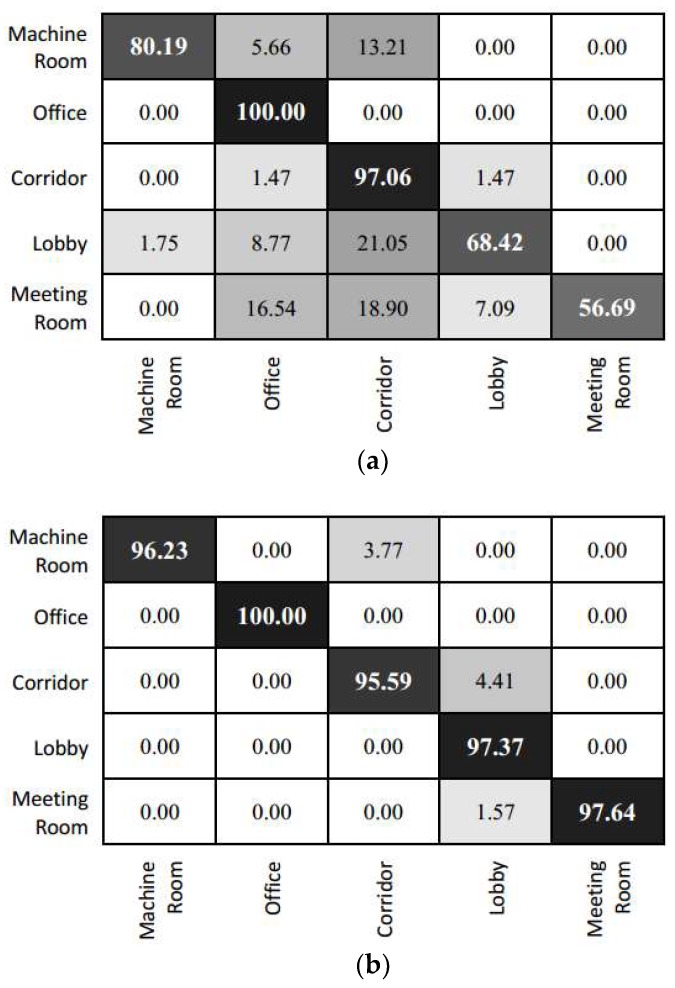
Confusion matrix of the prediction result for the laboratory scene. (**a**) Inception V3; (**b**) the feature fusion model.

As for the prediction result in individual categories, we can refer to the confusion matrix of the experimental result (as shown in Figure 11). The prediction of Inception V3 is highly efficient for the office and corridor categories. This is mainly because the features of these scenes are relatively simple. For instance, the corridor contains relatively fewer foreground objects. However, this model fails to capture prominent features due to the confusion in corridor and other categories. Images from other categories are easily misclassified as belonging to the office or corridor category. The feature fusion model yields a relatively average accuracy for each category. Given that the lobby is adjacent to several other types of scenes, a higher amount of test data is misclassified into the lobby category. The results of the feature fusion model show that error points are focused in the junction area of scenes with transparent surfaces (because of glass doors and so on.), such as the boundary between the corridor and the machine room or lobby. At these transparent junctions, significant errors might occur in image information. This might lead to the situation where the location and image information are biased and classified into a false category.

#### 5.1.2. The Result of Railway Station Scene

**Figure 12:**
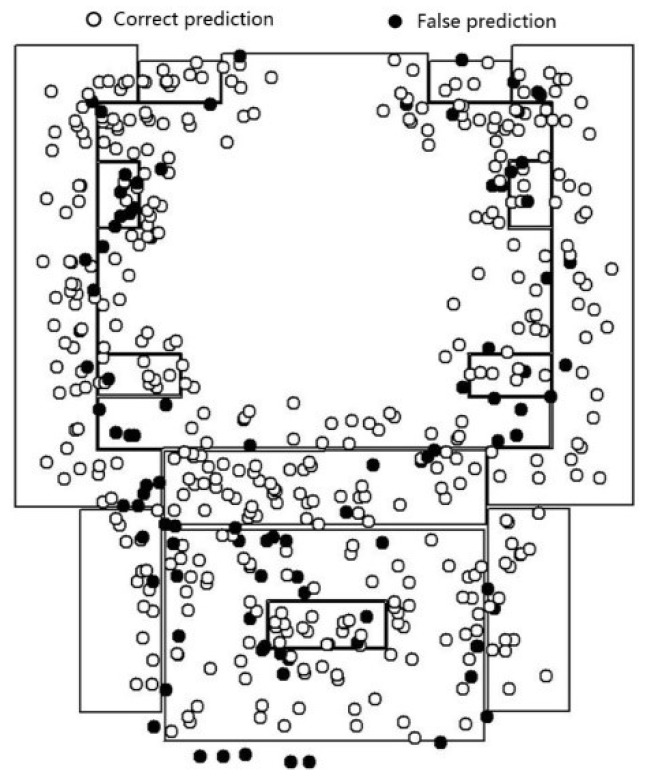
Overlapping result of location points in the railway station scene.

For the railway station scene, the fine-tuned Inception V3 model achieves convergence after 2000 iterations. The proposed model achieves convergence after 15,000 iterations. The test set is 15% of the simulative localization and image set. The results of the overlapping of the positioning point of the test sample set and scene boundary are shown in Figure 12. As the area of railway station scene is wider than that of the scene on the second floor of the laboratory, the effect of the positioning error is reduced. The proportion of the scene categories in the positioning points that are consistent with the real value is 81.2% and the error points are still mainly concentrated near the scene boundary.

**Figure 13:**
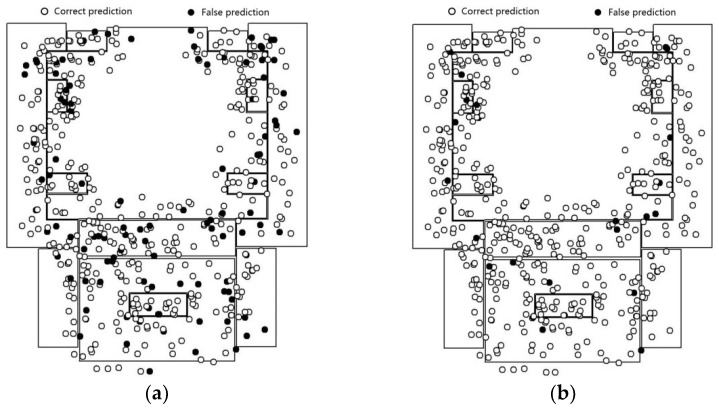
Prediction result for the railway station scenario. (**a**) Inception V3; (**b**) the feature fusion model.

The accuracy of the results using the fine-tuned Inception V3 model is 77.7%, and the distribution of the prediction results is shown in Figure 13a. In contrast to smaller indoor scenes (such as the laboratory), the proportion of the background in the railway station image has increased. Thus, a smaller scene area (such as the service counter and the security check channel) is easily affected by background information from scenes with larger areas at the railway station. The features of the images are biased towards background information of the larger area scene. Thus, the number of error points increase.

The accuracy of the railway station scenery using the feature fusion model is 96.6%. The distribution of predicted result is shown in Figure 13b. Compared with the fine-tuned Inception V3, owing to the combination of image features and positioning features, the description information of the scenes with smaller regions is more detailed, and the influence of the background information of a larger scene area is reduced. Hence, the occurrence of the situation in which a smaller area scene is incorrectly predicted is relatively less frequent.

**Figure 14:**
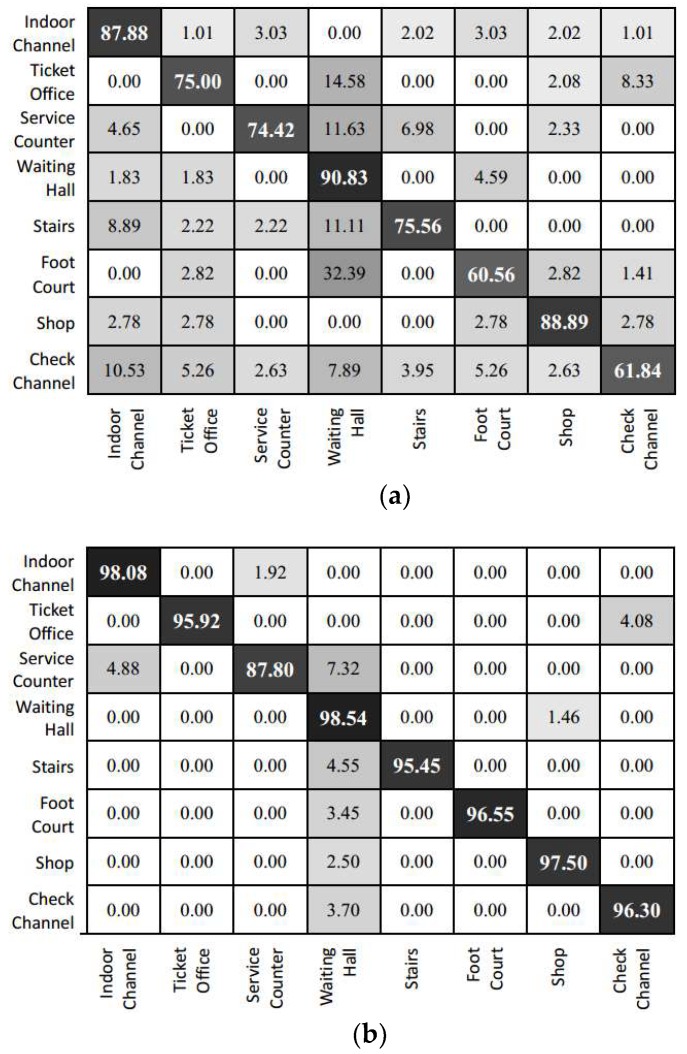
Confusion matrix of the prediction result in railway station. (**a**) Inception V3; (**b**) the feature fusion model.

Figure 14 shows the confusion table for the eight categories, namely indoor channel, ticket office, service counter, waiting hall, stairs, food court, shop, and security check channel. The results of the fine-tuned Inception V3 model show that the features of scenes with smaller areas are easily affected by scenes with larger areas (such as waiting hall and indoor channel) and rich background information. Therefore, more images are classified to the waiting hall and indoor channel categories. Meanwhile, the prediction for smaller area scenes (such as service counter, stairs, and check channel) is generally not good as those of the larger area scenes. Owing to the fusion of image features and positioning features, the feature fusion model in this paper has more detailed description information for scenes with small areas and weaker confusion effects from the background information of scenes of larger areas. Thus, the number of erroneous classifications for small areas is reduced. Furthermore, the results show that scenes of larger areas are predicted more accurately. Categories such as service counters and stairs, which is surrounded by larger scenes such as waiting rooms and indoor channels, are partly misclassified to larger area scene but it is reduced compared to the prediction result of the fine-tuned Inception V3 model.

### 5.2. Generalization Experiments

**Figure 15:**
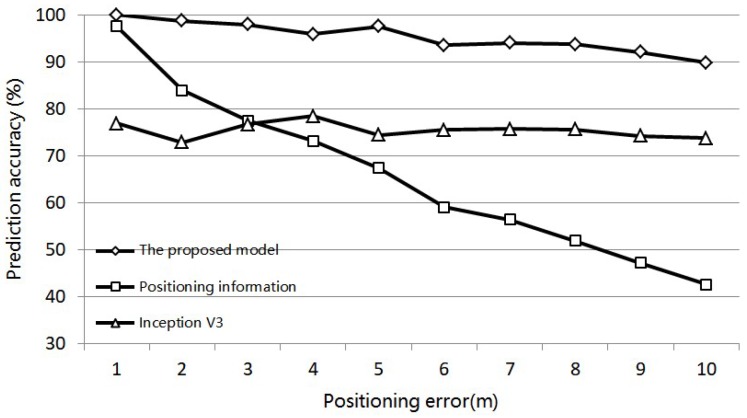
The prediction result of data set with different positioning error.

In order to verify the generalization ability of the feature fusion model in the condition with positioning data of different positioning accuracy, we change the positioning errors of the simulative positioning and image dataset from 1 m to 10 m with the corresponding images remained the same. The prediction result is shown in Figure 15.

As shown in the prediction results, by increasing the positioning error, the prediction accuracy based on positioning information decreases significantly to 40% when the error comes to 10 m. As for the feature fusion model, the image information can be used to compensate for the low accuracy of the positioning information. In the broken line of accuracy, as shown in Figure 15, it can be seen that the line representing the feature fusion model gets increasingly close to the line of the Inception V3 as the positioning error is increased. This means that greater weight is given to the image feature. The comparison of the results shows that, although the increase of error range causes a dramatic decrease in the accuracy of prediction solely with positioning information, the proposed model’s prediction accuracy decreases within a relatively small range. It can be proved that the feature-linking neural layers in the proposed model plays a role in the combined decision made using image and positioning information. Even though the positioning is made in indoor conditions, such as conditions when the signals of opportunity received by mobile devices are weak or under interference, the proposed model can increase prediction accuracy by applying positioning information as a supplementary feature to the image feature. Thus, the prediction has a stable accuracy and the model has appreciable generalization when confronted with positioning data of varying accuracy.

**Figure 16:**
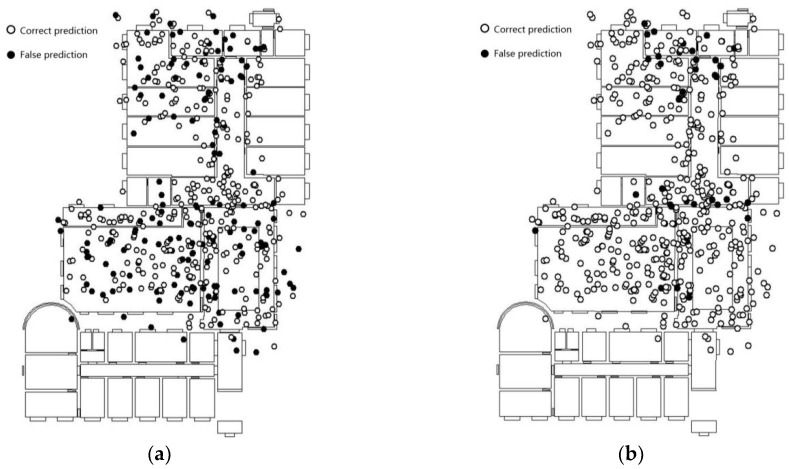
The prediction result in the 3rd floor of laboratory scenario. (**a**) Inception V3; (**b**) the feature fusion model.

To validate the generalization of the model with images from different scenes of similar categories, we chose the third floor of the laboratory, which has scenic similarities to the second floor of the laboratory, as the place to collect the validation set. This validation set contains 500 positioning points. We used the validation set to test the proposed model that was trained using the training set from the scenery of the second floor. The results show that the accuracy of the fine-tuned Inception V3 model, which was also trained using the dataset from the second floor (see Figure 16a), is 69.4%. Additionally, the accuracy of the feature fusion model (see Figure 16b) is 91.4%. The feature fusion model achieves better prediction results in recognizing other dataset apart from the training set, compared with the fine-tuned Inception V3 model.

The prediction results of the individual categories (shown in Table 3) shows that the accuracy of the feature fusion model has increased significantly compared to that of the fine-tuned Inception V3 model, especially with regard to scenery with wide areas, simple scenery features, or simple layouts. However, regarding the office scenery, owing to the differences between the office scene in the generalization test set and the office scene in the pre-training set, and the similarity of office scenery and other scenery in the training sets, the validation result is still not ideal. Therefore, the different functionality of similar scenes must be considered when constructing the categories for the scenery. Furthermore, the prediction accuracy of the feature fusion model is greater at the junction areas of scenes where the scenes can be seen through the junction boundary; the same as that of Inception V3 model, the junction area with transparent borders is still the main distribution area of the error points.

## 6. Conclusions

This paper proposed a deep learning scene recognition method based on localization enhancement. We use the method of deep learning to enhance the scenery features by fusing the scene level image feature and the positioning feature. The image feature is extracted from image data by a fine-tuned convolutional neural network, while the positioning feature is extracted from positioning information, in the form of the combination of the scene location feature and feature of the relationship with surrounding scenes. Experiments show that the proposed model has better accuracy in the environment with complex indoor scenes. Compared with the traditional convolution neural network, the proposed model has better generalization ability when dealing with scene image data of the same kind, but from different sources, and also works well in positioning data of different error ranges.

## Figures and Tables

**Table 1:** Main parameters set in the training process.

Parameter Type		Parameter Name	Recommended Value	Way to Adjust
Fine-tune Inception V3 model	Hyper-parameter	batch_size	100	Based on experience
		optimizer	Gradient decent	
		learning_rate	0.01	
		iteration	4000	
		testing_percentage	10%	
		validation_percentage	10%	
	Normal parameter	$W_{\mathrm{output}},b_{\mathrm{output}}$	Parameters of the fully connected fine-tuned layer	Training
Train the feature fusion and decision layers	Hyper-parameter	batch_size	32	Based on experience
		optimizer	Gradient decent	
		learning_rate	0.01	
		iteration	5000	
		testing_percentage	15%	
		validation_percentage	15%	
	Normal parameter	$W_{\mathrm{fuse}},b_{\mathrm{fuse}}$	Parameters of the fully connected feature fusion layer	Training
		$W_{\mathrm{output}},b_{\mathrm{output}}$	Parameters of the fully connected decision layer	

**Table 2:** Design of different experimental approaches.

Plan	Data Set	Tested Model	Method of Comparison
1	Training and test set of simulative positioning and image in the second floor of the laboratory	Fine-tune Inception V3 model The feature fusion model	Distribution of prediction result. Total prediction accuracy Confusion matrix of the prediction result.
2	Training and test set of simulative positioning and image at the railway station		
3	Training and test set with different positioning errors in the second floor of the laboratory	The feature fusion model	Total prediction accuracy Prediction accuracy for each category.
4	Test set in the third floor of the laboratory	Fine-tune Inception V3 model The feature fusion model	Distribution of prediction result. Total prediction accuracy. Confusion matrix of the prediction result

**Table 3:** Prediction result for each category.

	Scene	Machine Room	Office	Corridor	Lobby
Model					
Inception V3		66.8%	45.0%	87.7%	69.1%
The feature fusion model		94.8%	57.5%	97.7%	90.8%

## References

[1] Papapostolou A., Chaouchi H. (2011). Scene analysis indoor positioning enhancements. Ann. Telecommun..

[2] Gustafsson F., Gunnarsson F. (2005). Mobile positioning using wireless networks: Possibilities and fundamental limitations based on available wireless network measurements. IEEE Signal Process Mag..

[3] Chen R., Chen L. (2017). Indoor Positioning with Smartphones: The State-of-the-art and the Challenges. Acta Geod. Cartogr. Sin..

[4] Henderson J.M. (2005). Introduction to real-world scene perception. Vis. Cognit..

[5] Chen M., Gonzalez S., Vasilakos A., Cao H., Leung V.C. (2011). Body Area Networks: A Survey. Mobile Netw. Appl..

[6] Duda R.O., Hart P.E. (1973). Pattern Classification and Scene Analysis.

[7] Merry L.A., Faragher R.M., Scheding S. (2010). Comparison of opportunistic signals for localisation. IFAC Proc. Volumes.

[8] Yang C., Nguyen T., Venable D., White M., Siegel R. Cooperative position location with signals of opportunity. Proceedings of the IEEE 2009 National Aerospace & Electronics Conference (NAECON).

[9] Popleteev A. Indoor positioning using ambient radio signals: Data acquisition platform for a long-term study. Proceedings of the 13th Workshop on Positioning, Navigation and Communications (WPNC).

[10] Oliva A., Torralba A. (2001). Modeling the shape of the scene: A holistic representation of the spatial envelope. Int. J. Comput. Vis..

[11] Wang H., Gould S., Koller D. (2013). Discriminative Learning with Latent Variables for Cluttered Indoor Scene Understanding. Commun. ACM.

[12] Quattoni A., Torralba A. Recognizing indoor scenes. Proceedings of the IEEE Conference on Computer Vision and Pattern Recognition.

[13] Swadzba A., Wachsmuth S. Indoor scene classification using combined 3D and gist features. Proceedings of the Asian Conference on Computer Vision.

[14] Wu J., Rehg J.M. (2011). CENTRIST: A visual descriptor for scene categorization. IEEE Trans. Pattern Anal. Mach. Intell..

[15] Zhou B., Lapedriza A., Khosla A., Oliva A., Torralba A. (2017). Places: A 10 million image database for scene recognition. IEEE Trans. Pattern Anal. Mach. Intell..

[16] Krizhevsky A., Sutskever I., Hinton G.E. ImageNet classification with deep convolutional neural networks. Proceedings of the 25th International Conference on Neural Information Processing Systems.

[17] Szegedy C., Vanhoucke V., Ioffe S., Shlens J., Wojna W. Rethinking the inception architecture for computer vision. Proceedings of the IEEE Conference on Computer Vision and Pattern Recognition (CVPR).

[18] Nguyen L.D., Lin D., Lin Z., Cao J. Deep CNNs for microscopic image classification by exploiting transfer learning and feature concatenation. Proceedings of the 2018 IEEE International Symposium on Circuits and Systems (ISCAS).

[19] Tang P., Wang H., Kwong S. (2017). G-MS2F: GoogLeNet based multi-stage feature fusion of deep CNN for scene recognition. Neurocomputing.

[20] Oliva A., Torralba A. (2006). Building the gist of a scene: The role of global image features in recognition. Prog. Brain Res..

[21] Yuan Y., Mou L., Lu X. (2015). Scene recognition by manifold regularized deep learning architecture. IEEE Trans. Neural Netw. Learn. Syst..

[22] Zhou B., Lapedriza A., Xiao J., Torralba A., Oliva A. Learning deep features for scene recognition using places database. Proceedings of the 27th International Conference on Neural Information Processing Systems.

[23] Deng J., Dong W., Socher R., Li L.-J., Li K., Li F.-F. Imagenet: A large-scale hierarchical image database. Proceedings of the 2009 IEEE Conference on Computer Vision and Pattern Recognition.

[24] Oquab M., Bottou L., Laptev I., Sivic J. Learning and transferring mid-level image representations using convolutional neural networks. Proceedings of the IEEE Conference on Computer Vision and Pattern Recognition.

[25] Lin T.-Y., Belongie S., Hays J. Cross-view image geolocalization. Proceedings of the IEEE Conference on Computer Vision and Pattern Recognition.

[26] Baatz G., Saurer O., Köser K., Pollefeys M. (2012). Large scale visual geo-localization of images in mountainous terrain. Computer Vision-ECCV 2012.

[27] Berg T., Liu J., Lee S.W., Alexander M.L., Jacobs D.W., Belhumeur P.N. In Birdsnap: Large-scale fine-grained visual categorization of birds. Proceedings of the IEEE Conference on Computer Vision and Pattern Recognition (CVPR).

[28] Tang K., Paluri M., Fei-Fei L., Fergus R., Bourdev L. Improving image classification with location context. Proceedings of the IEEE International Conference on Computer Vision.

[29] Ardeshir S., Zamir A.R., Torroella A., Shah M. (2014). GIS-Assisted Object Detection and Geospatial Localization. Lecture Notes in Computer Science, Proceedings of the 13th European Conference on Computer Vision, Zurich, Switzerland, 6–12 September 2014.

[30] Amlacher K., Fritz G., Luley P., Almer A., Paletta L. Geo-contextual priors for attentive urban object recognition. Proceedings of the IEEE International Conference on Robotics and Automation.

[31] Yu J., Luo J. Leveraging probabilistic season and location context models for scene understanding. Proceedings of the 2008 International Conference on Content-Based Image and Video Retrieval.

[32] Yan B., Janowicz K., Mai G., Zhu R. xNet+ SC: Classifying Places Based on Images by Incorporating Spatial Contexts. Proceedings of the 10th International Conference on Geographic Information Science (GIScience 2018).

[33] Szegedy C., Liu W., Jia Y., Sermanet P., Reed S., Anguelov D., Erhan D., Vanhoucke V., Rabinovich A. Going deeper with convolutions. Proceedings of the IEEE Conference on Computer Vision and Pattern Recognition (CVPR).

[34] Ioffe S., Szegedy C. Batch Normalization: Accelerating Deep Network Training by Reducing Internal Covariate Shift. https://arxiv.org/abs/1502.03167.

[35] Rasmus A., Valpola H., Honkala M., Valpola H., Raiko T. Semi-Supervised Learning with Ladder Networks. Proceedings of the Advances in Neural Information Processing Systems 28 (NIPS 2015).

[36] Simonyan K., Zisserman A. Very Deep Convolutional Networks for Large-Scale Image Recognition. Comput. Vis. Pattern Recognit..

[37] Pan S.J., Yang Q. (2010). A survey on transfer learning. IEEE Trans. Knowl. Data Eng..

[38] Martin A., Ashish A., Paul B., Brevdo E., Chen Z., Citro C., Corrado G.S., Davis A., Dean J., Devin M. Tensorflow: Large-Scale Machine Learning on Heterogeneous Distributed Systems. https://arxiv.org/abs/1603.04467.

